# Supplementary material for: Reporting guidelines in medical artificial intelligence: a systematic review and meta-analysis
Source: Commun Med (Lond). 2024 Apr 11;4:71. doi: 10.1038/s43856-024-00492-0 (PMC11009315; doi:10.1038/s43856-024-00492-0)
Supplement: Supplementary file 2 — Supplementary Information [file 43856_2024_492_MOESM2_ESM.pdf]

Reporting Guidelines in Medical Artificial Intelligence: A Systematic Review and Meta-Analysis

Supplementary Information

Fiona R. Kolbinger (1, 2, 3, 4, *), Gregory P. Veldhuizen (1, *), Jiefu Zhu (1), Daniel Truhn (5), Jakob Nikolas Kather (1, 6, 7, 8, +)

1 Else Kroener Fresenius Center for Digital Health, Technical University Dresden, Dresden, Germany
2 Department of Visceral, Thoracic and Vascular Surgery, University Hospital and Faculty of Medicine Carl Gustav Carus, Technical University Dresden, Dresden, Germany
3 Weldon School of Biomedical Engineering, Purdue University, West Lafayette, Indiana, USA
4 Regenstrief Center for Healthcare Engineering, Purdue University, West Lafayette, Indiana, USA
5 Department of Diagnostic and Interventional Radiology, University Hospital Aachen, Germany
6 Department of Medicine III, University Hospital RWTH Aachen, Aachen, Germany
7 Department of Medicine I, University Hospital Dresden, Dresden, Germany
8 Medical Oncology, National Center for Tumor Diseases (NCT), University Hospital Heidelberg, Heidelberg, Germany

* contributed equally

+ Correspondence to: Prof. Dr. Jakob Nikolas Kather, Else Kroener Fresenius Center for Digital Health, Technical University Dresden, Germany, Email: jakob-nikolas.kather@alumni.dkfz.de

Supplementary Table 1: Guideline items included in published guidelines regulating medical AI research. Each included guideline was evaluated for the purpose of determining which were either fully, partially, or not covered by each publication individually. Aspects that were directly described in a guideline including some details or examples were considered "fully" covered, aspects mentioned implicitly using general terms were considered "partially" covered. Abbreviations and symbols: Inclusion process: Guideline identified via a systematic, blinded review of the literature (PubMed, EQUATOR Network library of reporting guidelines) (*), Guideline identified by additional pre-specified inclusion procedure (+), Guideline added after incidental finding (#), Guideline on reporting of predictive models with AI-specific guideline under development (%). Guideline Type: General (G), Subject-specific (S). Level of consensus: Comprehensive (H), Collaborative (M), Expert-led (L). Guideline item coverage: Fully covered (Y), Partially covered (P), Not covered (N).

	Guideline Item	Description (specification and/or example content)	Item type	AI-specific item?	STARE-HI	TRIPOD	Luo et al.	CONSORT-AI	SPIRIT-AI	Schwendicke et al.	CLEAR Derm	DECIDE-AI	CLEAR	% Items included in comprehensive guidelines	Good ML Practice	MI-CLAIM	PRIME	DOME	Shen	Hatt et al.	% Items included in collaborative guidelines	Vihinen	CLAIM	MINIMAR	Stevens et al.	CAIR	PIECES	Zukotynski et al.	El Naqa et al.	Jones et al.	R-AI-DIOLOGY	Volovici et al.	% Items included in expert-led guidelines (Y/P)	% Item included in all guidelines (Y/P)	% Items included in general guidelines (Y/P)	% Items included in specific guidelines (Y/P)
**Year**					2009	2015	2016	2020	2020	2021	2022	2022	2023		2019	2020	2020	2021	2022	2023		2012	2020	2020	2020	2021	2021	2021	2021	2022	2022	2022				
**Inclusion Process**					"+"	%	*	*	*	*	*	*	*		"+"	*	*	*	*	#		*	#	*	*	*	#	*	#	*	*	#				
**Guideline Type**					G	G	G	G	G	S	S	G	S		G	G	S	G	S	S		G	S	G	G	G	S	S	S	S	S	G				
**Level of Consensus**					H	H	H	H	H	H	H	H	H		M	M	M	M	M	M		L	L	L	L	L	L	L	L	L	L	L				
**Clinical Rationale**	Topic	Predictive AI	Content	Yes	N	Y	Y	Y	Y	Y	N	Y	Y	0.78	N	N	P	N	N	N	0.17	N	Y	N	N	Y	N	N	N	Y	N	N	0.27	0.42	0.43	0.50
	Study Design	Retrospective vs. prospective, prognostic vs. diagnostic	Content	Partially	Y	Y	Y	N	N	Y	N	Y	Y	0.67	N	Y	Y	N	N	Y	0.50	N	Y	N	N	N	N	N	N	N	N	N	0.09	0.38	0.36	0.50
	Prediction Problem	Prediction target, outcome parameters, performance metrics	Content	Yes	Y	Y	Y	Y	Y	Y	N	Y	Y	0.89	P	Y	Y	N	N	Y	0.67	N	Y	P	P	P	Y	N	N	Y	P	N	0.64	0.73	0.79	0.75
	Clinical Setting	Details on the clinical problem and intended use	Content	No	Y	Y	Y	Y	Y	Y	Y	Y	Y	1.00	Y	Y	N	N	P	Y	0.67	N	Y	P	P	Y	Y	N	P	Y	Y	N	0.73	0.81	0.79	0.83
	Rationale	Relation between prediction problem and clinical goal	Content	Yes	Y	P	Y	Y	Y	P	P	Y	P	1.00	Y	Y	N	N	N	Y	0.50	N	Y	N	P	P	P	N	P	Y	P	N	0.64	0.73	0.71	0.75
	Existing AI and Statistical Models	Performance metrics, level of translation, clinical application	Content	Yes	P	Y	Y	N	Y	Y	N	P	Y	0.78	N	P	Y	Y	N	N	0.50	Y	N	N	N	N	P	N	P	P	P	Y	0.55	0.62	0.64	0.58
	State-of-the-art	Identify state-of-the-art clinical solution and use as a baseline for comparison	Quality	Partially	P	P	N	N	N	P	N	N	P	0.44	P	Y	N	Y	P	N	0.67	P	N	N	N	N	N	N	N	N	N	N	0.09	0.35	0.43	0.33
**Data**	Data Sources, Types, and Structure	Original data format and volume, facility details, structured vs. unstructured data	Content	Partially	Y	Y	Y	P	P	Y	Y	Y	Y	1.00	P	Y	Y	P	P	Y	1.00	N	Y	Y	Y	Y	Y	Y	Y	Y	Y	Y	0.91	0.96	0.93	1.00
	Data Selection	Inclusion and exclusion criteria at the level of data and participants	Content	Partially	Y	Y	Y	Y	Y	Y	N	Y	Y	0.89	P	N	N	N	P	P	0.50	N	Y	Y	N	Y	Y	N	N	Y	N	Y	0.55	0.65	0.71	0.67
	Data Preprocessing	Data transformation, handling of missing data and outliers	Content	Yes	Y	Y	Y	Y	Y	Y	Y	Y	Y	1.00	P	Y	Y	N	P	Y	0.83	N	Y	N	Y	Y	Y	N	Y	P	Y	Y	0.73	0.85	0.79	0.92
	Labeling of Input Data	Clinical outcome vs. expert rating, number and expertise of labellers	Content, Quality	Partially	Y	N	N	Y	Y	Y	P	Y	Y	0.78	Y	N	N	N	P	Y	0.50	N	Y	P	P	P	Y	N	Y	Y	N	P	0.73	0.69	0.64	0.75
	Rater Variability	Inter- and intrarater variability	Quality	Partially	Y	N	N	N	N	Y	N	N	Y	0.33	N	N	N	N	N	N	0.00	N	Y	N	N	Y	N	N	P	N	N	N	0.27	0.23	0.14	0.33
	Data Processing Location	Specification of data processing location (local vs. cloud, external institutions involved in data processing, data flow)	Content	Partially	N	N	N	N	N	Y	N	N	N	0.11	N	N	N	N	Y	N	0.17	N	N	N	N	N	N	N	N	N	Y	N	0.09	0.12	0.00	0.33
	De-Identification	Address anonymization/de-identification of data	Quality	Partially	N	N	N	N	N	Y	N	N	Y	0.22	N	N	N	N	Y	N	0.17	N	Y	N	N	N	Y	N	N	N	Y	N	0.27	0.23	0.00	0.58
	Data Dictionary	Release data dictionary with explanations of variables	Content	Partially	N	N	N	N	N	N	N	N	N	0.00	P	N	Y	N	N	N	0.33	N	Y	N	N	N	N	N	N	N	N	N	0.09	0.12	0.07	0.25
	Data Leakage	Independence of training/validation/test data (i.e. do not use evaluation sets for feature selection, preprocessing steps or parameter tuning)	Quality	Yes	N	N	N	N	N	N	N	N	P	0.11	Y	Y	N	Y	N	Y	0.67	Y	N	N	P	N	Y	N	P	Y	N	N	0.45	0.38	0.36	0.42
	Representativeness	Training and test data should be representative of real-world clinical settings	Quality	Yes	P	P	N	N	N	Y	N	N	N	0.33	Y	Y	N	Y	P	P	0.83	N	N	N	N	N	N	N	Y	Y	P	Y	0.36	0.46	0.43	0.50
	Basic Statistics of the Dataset	Distribution of input and outcomes	Content	Partially	Y	Y	Y	N	N	N	Y	N	N	0.44	Y	N	Y	Y	N	N	0.50	N	Y	Y	N	N	N	N	Y	Y	N	Y	0.45	0.46	0.50	0.42
**Model Training and Validation**	Type of Prediction Model	Type of algorithm, classification vs. regression	Content	Yes	N	Y	Y	Y	Y	Y	N	Y	Y	0.78	N	P	Y	Y	N	N	0.50	N	Y	Y	N	Y	Y	Y	Y	Y	N	N	0.64	0.65	0.64	0.67
	Model Development	Identification and removal of redundant independent variables, model training and selection strategy	Content	Yes	N	Y	Y	N	P	Y	Y	P	Y	0.78	P	Y	Y	Y	P	P	1.00	Y	Y	Y	Y	Y	Y	P	Y	Y	P	P	1.00	0.92	0.86	1.00
	Model Validation	Internal vs. external vs. cross validation, validation metrics	Content	Yes	Y	Y	Y	N	N	Y	P	P	Y	0.78	P	P	Y	Y	N	P	0.83	Y	Y	P	Y	Y	Y	Y	Y	Y	P	P	1.00	0.88	0.86	0.92
	Model Interpretability	Statement on model interpretability	Content, Quality	Yes	N	N	Y	N	N	Y	N	N	Y	0.33	Y	N	Y	Y	N	N	0.50	N	Y	N	P	N	N	N	P	Y	N	Y	0.45	0.42	0.36	0.50
	Model Performance and Interpretation	Outcome metrics, confidence intervals	Content	Yes	Y	Y	Y	P	P	P	Y	Y	Y	1.00	Y	P	P	Y	P	N	0.83	Y	Y	Y	Y	P	Y	N	Y	Y	P	P	0.91	0.92	1.00	0.83
	Computational Cost	Model execution time, floating point operations per second	Content	Yes	N	N	N	N	N	N	N	N	N	0.00	N	N	N	Y	N	N	0.17	N	N	N	N	N	N	Y	P	N	N	N	0.18	0.12	0.07	0.17
	Statistical Methods	Appropriate methods and significance levels for performance comparison of baseline and proposed model	Quality	Partially	Y	Y	N	N	N	Y	N	N	Y	0.44	Y	Y	N	Y	N	N	0.50	P	Y	N	N	N	P	N	Y	Y	N	Y	0.55	0.50	0.50	0.50
	Performance Errors	Identification and analysis of errors	Content, Quality	Yes	Y	Y	N	Y	Y	N	P	Y	P	0.78	Y	N	P	N	P	N	0.50	P	Y	N	N	Y	N	N	N	N	N	P	0.36	0.54	0.64	0.50
	Over-/Underfitting	Assessment of the possibility of over-/underfitting (i.e. by reporting indicators such as train vs. test error)	Quality	Yes	N	N	N	N	N	N	N	N	N	0.00	Y	N	N	Y	N	N	0.33	P	N	N	P	N	N	N	Y	N	N	N	0.27	0.19	0.29	0.08
**Critical Appraisal**	Clinical Implications and Practical Value	Potential augmentations of clinical workflows, potential changes in clinical decision making	Content	Partially	Y	Y	Y	Y	Y	Y	Y	Y	Y	1.00	Y	N	N	N	Y	N	0.33	N	Y	N	N	N	N	N	Y	N	Y	N	0.27	0.54	0.50	0.58
	Translation	Details on integration into clinical workflow	Content	Partially	N	Y	N	N	N	N	N	Y	N	0.22	Y	N	N	N	P	N	0.33	N	Y	N	N	N	N	N	N	Y	Y	N	0.27	0.27	0.21	0.42
	Limitations	Bias, generalizability, interpretation pitfalls	Content	Partially	Y	Y	Y	N	N	P	Y	Y	Y	0.78	Y	N	Y	N	P	N	0.50	N	Y	N	N	N	N	N	Y	P	N	Y	0.36	0.54	0.43	0.67
	Data Publication	Publication of datasets or inclusion of a statement on public availability	Content	Partially	N	Y	N	N	N	Y	N	Y	Y	0.44	N	Y	N	Y	N	N	0.33	Y	N	Y	Y	Y	N	N	N	Y	N	Y	0.55	0.46	0.64	0.33

Ethics and Reproducibility	Code Publication	Publication of code or inclusion of a statement on public availability	Content	Yes	N	Y	N	Y	Y	Y	N	Y	Y	0.67	N	Y	Y	Y	N	N	0.50	N	N	Y	Y	Y	N	P	Y	N	N	Y	0.55	0.58	0.71	0.42
	AI Intervention Publication	Publication of AI Intervention or inclusion of a statement on public availability	Content	Yes	P	N	N	Y	Y	N	N	N	Y	0.44	N	Y	N	Y	N	N	0.33	N	N	N	N	N	N	N	N	N	N	N	0.00	0.23	0.36	0.17
	Future Updates	Details on future software/algorithm updates (i.e. how users will be informed)	Content	Partially	N	N	N	N	N	N	N	N	N	0.00	P	N	N	N	P	N	0.33	N	N	N	N	N	P	N	N	Y	Y	N	0.27	0.19	0.07	0.42
	Ethical Statement	Details on IRB approval and informed consent procedure	Content	No	Y	Y	Y	N	N	Y	N	Y	Y	0.67	N	N	N	N	Y	N	0.17	N	N	N	N	Y	N	N	Y	N	N	N	0.18	0.35	0.36	0.33
	Equity and Access	Statement on equity, diversity and access to AI application	Content, Quality	Yes	N	N	N	N	N	P	N	P	P	0.33	P	N	N	N	Y	N	0.33	N	N	N	N	N	N	N	N	N	N	N	0.00	0.19	0.14	0.33
	Legal and Regulatory Aspects	Statement on legal and regulatory aspects	Content	Partially	N	N	N	N	N	Y	N	N	N	0.11	N	N	N	N	Y	N	0.17	N	N	N	N	N	N	N	N	Y	Y	N	0.18	0.15	0.00	0.42
